# Supplementary material for: Mycobacterium tuberculosis H2S Functions as a Sink to Modulate Central Metabolism, Bioenergetics, and Drug Susceptibility
Source: Antioxidants (Basel). 2021 Aug 13;10(8):1285. doi: 10.3390/antiox10081285 (PMC8389258; doi:10.3390/antiox10081285)
Supplement: Supplementary file 1 [file antioxidants-10-01285-s001.zip › antioxidants-1272948-supplementary.pdf]

Supplementary Materials:

Mycobacterium tuberculosis H_2S Functions as a Sink to Modulate Central Metabolism, Bioenergetics, and Drug Susceptibility

Tafara T. R. Kunota [1,†], Md. Aejazur Rahman [1,†], Barry E. Truebody [1], Jared S. Mackenzie [1], Vikram Saini [2], Dirk A. Lamprecht [1,‡], John H. Adamson [1], Ritesh R. Sevalkar [3], Jack R. Lancaster Jr. [4], Michael Berney [5], Joel N. Glasgow [3] and Adrie J. C. Steyn [1,3,6,*]

[1]Africa Health Research Institute, University of KwaZulu Natal, Durban, 4001, South Africa

[2]Department of Biotechnology, All India Institute of Medical Sciences, New Delhi -110029, India

[3]Department of Microbiology, University of Alabama at Birmingham, Birmingham, AL 35294, USA

[4]Department of Pharmacology and Chemical Biology and Vascular Medicine Institute, University of Pittsburgh School of Medicine, Pittsburgh, PA 15261, USA

[5]Department of Microbiology and Immunology, Albert Einstein College of Medicine, New York, NY 10464, USA

[6]Center for AIDS Research and Center for Free Radical Biology, University of Alabama at Birmingham, Birmingham, AL 35294, USA

† Co-first authors, both authors contributed equally.

‡ Current address: Janssen Pharmaceutica, Global Public Health, Turnhoutseweg 30, B-2340 Beerse, Belgium

* Correspondence: asteyn@uab.edu

Supplementary Figures and Tables

Supplementary Figure S1. *Mtb* H_2S production when exposed to exogenous Cys.
Supplementary Figure S2. Role of *Mtb* Rv1077 (CBS) in H_2S production.
Supplementary Figure S3. Multiple sequence alignment of the CDS/CBS protein family.
Supplementary Figure S4. Complete Rv3684/Cds1 amino acid sequence.
Supplementary Figure S5. In-gel BC assay of purified Cds1 and confirmation of *cds1* deletion in *Mtb* strains.
Supplementary Figure S6. Survival of *cds1*-deficient *Mtb* in macrophages.

Supplementary Figure S7. *Mtb* Δ*cds1* growth in the presence of fatty acids or precursors as a single carbon source.
Supplementary Figure S8. *Mtb* H_2S production when cultured in the presence of fatty acids or precursors as a single carbon source.
Supplementary Figure S9. Role of Cbs (Rv1077) in *Mtb* respiration.
Supplementary Figure S10. AOAA inhibits Cys-mediated increases in *Mtb* respiration.
Supplementary Figure S11. Exogenous H_2S reverses the respiratory defect in *Mtb* Δ*cds1* cells.
Supplementary Figure S12. SDS-PAGE of purified *O*-acetylserine sulfhydrylase (OASS).
Supplementary Figure S13. Cds1 regulates amino acid metabolism in *Mtb*.
Supplementary Figure S14. Gating strategy for detection of DHE-positive *Mtb* cells for measuring ROI.
Supplementary Figure S15. Mycothiol and ergothioneine levels in *Mtb* after exposure to CHP.

Supplementary Table S1. Bacterial strains used in this study.
Supplementary Table S2. Plasmids used in this study.
Supplementary Table S3. Oligonucleotides used in this study.
Supplementary Table S4. *Mtb* H37Rv enzymes putatively involved in sulfur-containing amino acid biosynthesis, H_2S production or sulfur metabolism.

REFERENCES

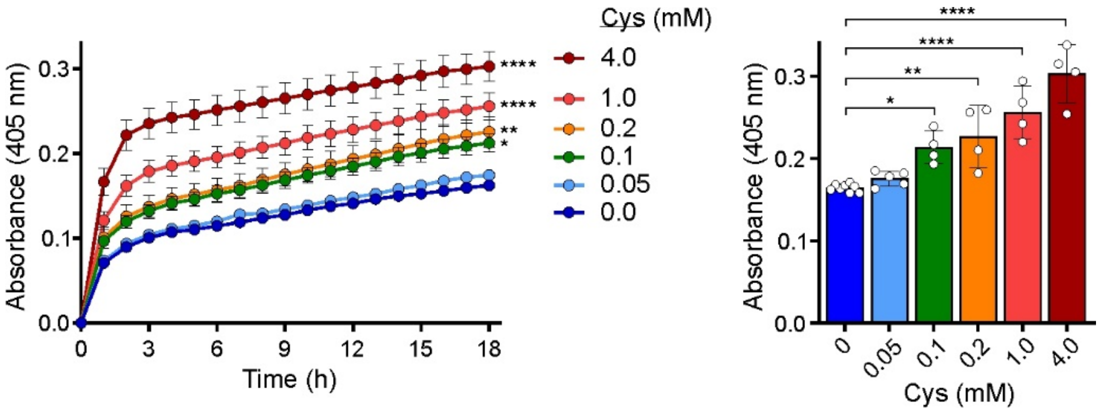

Supplementary Figure S1. *Mtb* H_2S production when exposed to exogenous Cys. The BC assay was used to measure H_2S production in *Mtb* when exposed to increasing concentrations of exogenous Cys (0-4 mM). The kinetics of H_2S production is shown in the left panel, whereas endpoint levels after 18 hours are shown in the right panel. Note that *Mtb* does not require addition of exogenous Cys to produce H_2S (see 0 mM Cys) and Figure 1. Data shown represents the mean ± SEM for 4 – 5 biological replicates. Statistical analysis was performed using GraphPad Prism 8.4.3. Two-way ANOVA with Dunnett's multiple comparisons test was used to determine statistical significance compared to 0.0 mM Cys. *$P < 0.05$, **$P < 0.01$, ****$P < 0.0001$.

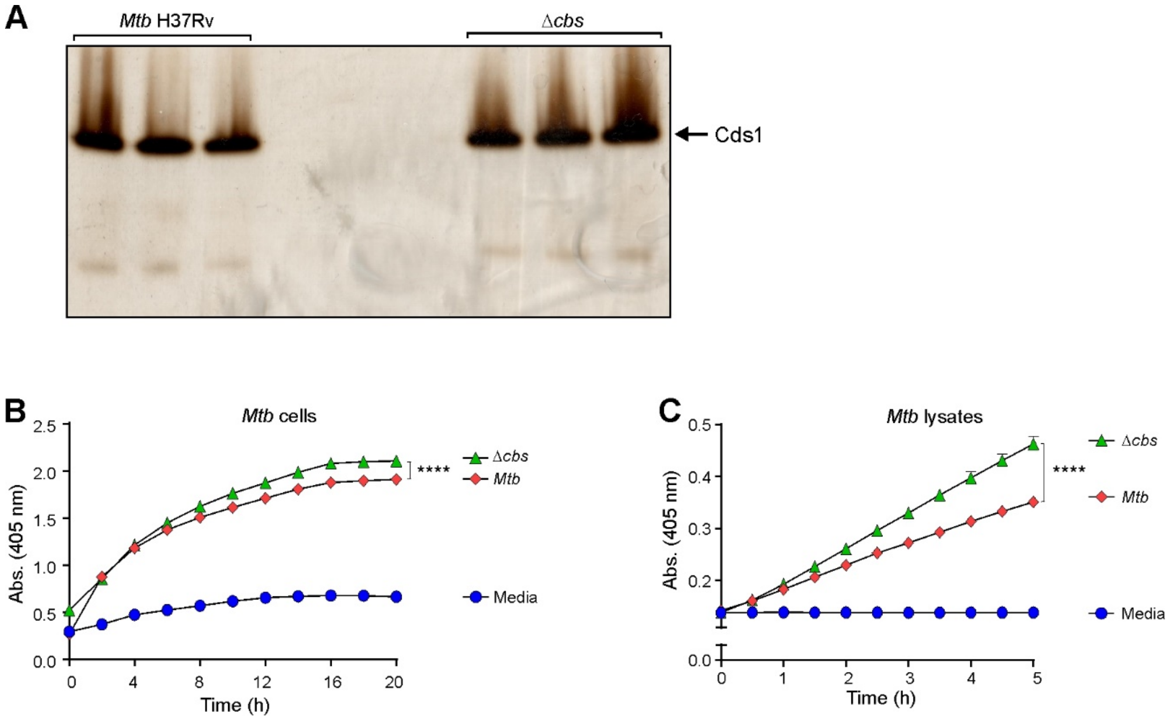

Supplementary Figure S2. Role of *Mtb* Rv1077 (CBS) in H_2S production. (A) Lysates of *Mtb* H37Rv and the *Mtb rv1077/cbs* deletion strain (Δ*cbs*) were separated on a native polyacrylamide gel and assayed for H_2S production using the in-gel BC assay. Arrow indicates the major H_2S producing enzyme, (n = 3). Time course of H_2S production in **(B)** intact *Mtb* H37Rv and Δ*cbs* cells, (n = 8) and **(C)** cell lysates in the presence of 20 mM Cys using the BC assay (n = 8). Representative experiments are shown. Each experiment was repeated independently at least twice. Data represent the mean ± SD. Statistical analysis was performed using GraphPad Prism 8.4.3. One-way ANOVA with Dunnett's multiple comparisons test was used to determine statistical significance. ****$P < 0.0001$.

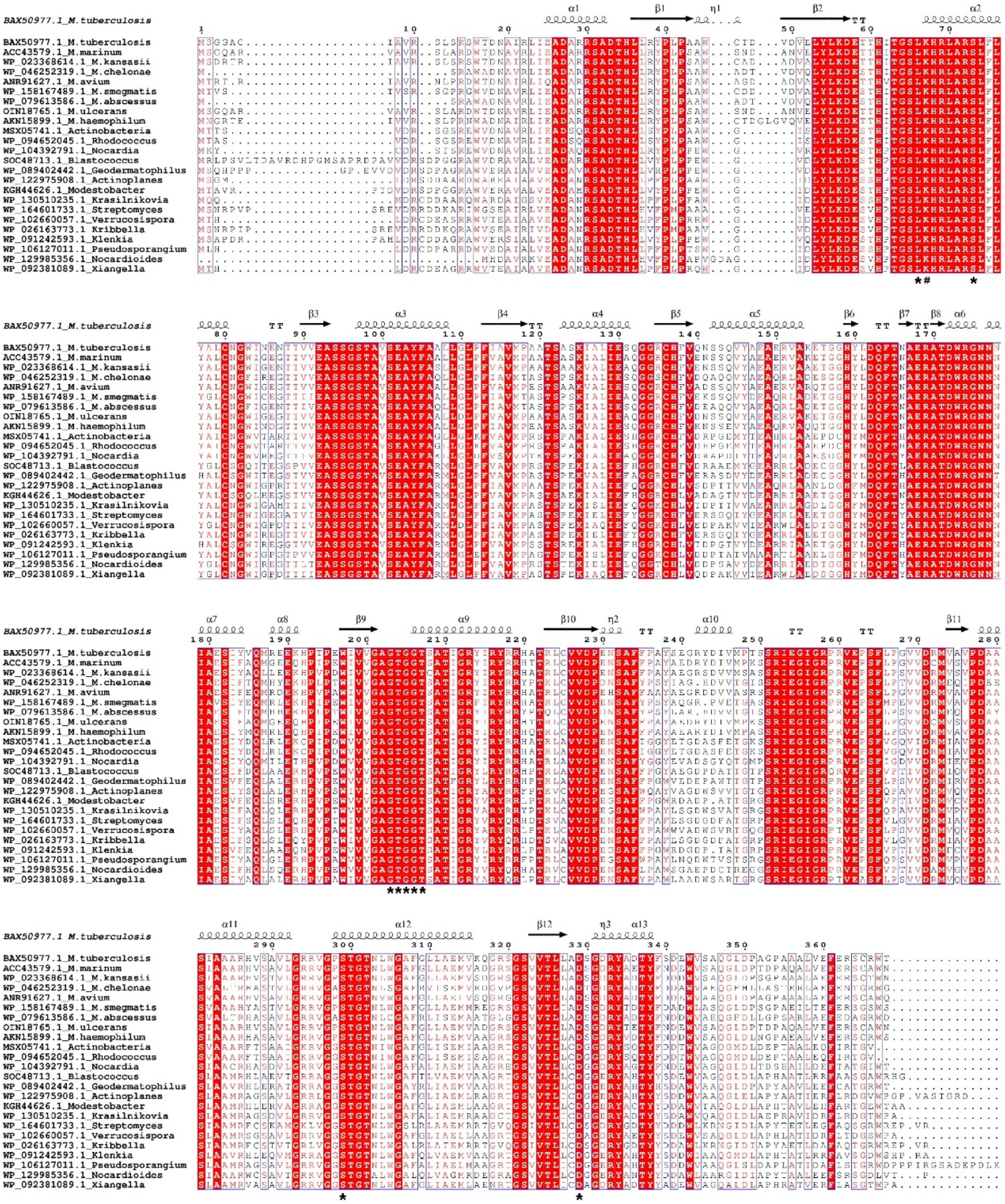

Supplementary Figure S3. Multiple sequence alignment of the CDS/CBS protein family. Multiple sequence alignment of Cds1 BLASTp. Structural alignment performed using T-Coffee online software[1] and rendered with predicted secondary structures using the ESPript. 3.0 server[2]. Identical amino acids are shaded in red. Asterisks (*) indicate residues of the predicted, conserved PLP binding domain (L^{66}, S^{73}, G^{203}, T^{204}, G^{205}, G^{206}, T^{207}, S^{299}, D^{329}). # indicates the predicted catalytic residue (K^{67}) which is conserved across orthologues with an identity >65%**.** Sequences were obtained from a BLASTp search of the NCBI database (https://blast.ncbi.nlm.nih.gov/Blast.cgi) using the primary sequence of Cds1 as identified by LC-MS/MS (Figure. 2c). CDS; cysteine desulfhydrase, CBS; cystathionine β-synthase.

A

N-terminal peptides detection in our study using LC MS/MS:

Sequence	# PSMs	Accessions No.	MH+ [Da]	Abundance
MSGGACIAVRSLSRSWTDNAIR	1	A0A089QT58	2408.20237	Low
SWTDNAIRLIEADAR	1	A0A089QT58	1730.86492	Low
SLSRSWTDNAIRLIEADAR	2	A0A089QT58	2174.11045	Low
SLSRSWTDNAIR	3	A0A089QT58	1405.71416	Low
SWTDNAIRLIEADARR	1	A0A089QT58	1886.99063	Low

B

Full length gene and protein sequence of Rv3684:

```
  P   T   V   S   V   R   -  ← Rv3683
 ccg aca gtg tcg gtg cgt tga
                  Rv3684 → ttg agc ggc ggg gcc tgt atc gcg gtc cgc agc cta tcc cgg agc tgg acg gac aac
                            M   S   G   G   A   C   I   A   V   R   S   L   S   R   S   W   T   D   N
gcg atc cgg ttg atc gag gcg gac gcc cgc cgt agc gcc gac acc cac ctg ctg cgc tac cca ctg ccc gct gcc
 A   I   R   L   I   E   A   D   A   R   R   S   A   D   T   H   L   L   R   Y   P   L   P   A   A
tgg tgc acg gat gtc gac gtc gag ctg tac ctc aag gac gag acg acc cat atc acc ggc agt ctc aaa cac cgg
 W   C   T   D   V   D   V   E   L   Y   L   K   D   E   T   T   H   I   T   G   S   L   K   H   R
ttg gca cgt tcg ttg ttc ctc tat gcg cta tgc aac ggc tgg atc aac gag aac acc acg gtg gtg gag gca tcg
 L   A   R   S   L   F   L   Y   A   L   C   N   G   W   I   N   E   N   T   T   V   V   E   A   S
tcg ggt tca acg gcg gtg tcc gag gcc tat ttc gcg gcg ctg ctg ggt ctg ccg ttc atc gcc gtg atg ccg gcc
 S   G   S   T   A   V   S   E   A   Y   F   A   A   L   L   G   L   P   F   I   A   V   M   P   A
gcg acc agc gct tcc aaa atc gcg ttg atc gaa tca caa ggt ggc cgt tgt cat ttc gtc cag aat tca agt caa
 A   T   S   A   S   K   I   A   L   I   E   S   Q   G   G   R   C   H   F   V   Q   N   S   S   Q
gtg tac gcc gag gcg gag cgc gtc gcc aag gaa acc ggc ggc cac tat ctg gac cag ttc acc aac gcg gag cgc
 V   Y   A   E   A   E   R   V   A   K   E   T   G   G   H   Y   L   D   Q   F   T   N   A   E   R
gca acc gac tgg cgc ggc aac aac aac atc gcc gag tcg atc tac gtg caa atg cgc gaa gag aag cac ccc acc
 A   T   D   W   R   G   N   N   N   I   A   E   S   I   Y   V   Q   M   R   E   E   K   H   P   T
ccg gaa tgg atc gtc gtg ggt gcg ggc acc ggc gga acc agc gcg acg atc ggc cgc tac atc cgc tac cga cgg
 P   E   W   I   V   V   G   A   G   T   G   G   T   S   A   T   I   G   R   Y   I   R   Y   R   R
cac gcg acc cgg ctg tgc gtc gtc gat ccg gag aat tcc gcg ttc ttc ccc gcg tac tcc gaa ggc cgg tac gac
 H   A   T   R   L   C   V   V   D   P   E   N   S   A   F   F   P   A   Y   S   E   G   R   Y   D
atc gtc atg ccc aca tcg tcc cgt atc gag ggc atc ggc cgg ccg cgg gtc gag ccg tcg ttt ctg ccc ggt gtg
 I   V   M   P   T   S   S   R   I   E   G   I   G   R   P   R   V   E   P   S   F   L   P   G   V
gtc gac cgc atg gtg gcg gtc ccc gac gcg gcg tcg atc gct gcc gcc cgg cat gtc agc gcc gtt ctg ggg cgc
 V   D   R   M   V   A   V   P   D   A   A   S   I   A   A   A   R   H   V   S   A   V   L   G   R
cga gtg gga ccg tct acc ggc acc aac ctc tgg ggc gcg ttc gga ctg ctc gcc gag atg gtc aag cag ggc cgc
 R   V   G   P   S   T   G   T   N   L   W   G   A   F   G   L   L   A   E   M   V   K   Q   G   R
agc ggc tcg gtg gtc aca ctg ctc gcc gac agc ggc gat cgc tac gcc gac acc tac ttt tcc gac gag tgg gtc
 S   G   S   V   V   T   L   L   A   D   S   G   D   R   Y   A   D   T   Y   F   S   D   E   W   V
agt gcc cag ggg ctc gat ccg gcc ggg ccg gct gcg gcg ctg gtg gaa ttc gag cgc tcc tgt cga tgg acg tga
 S   A   Q   G   L   D   P   A   G   P   A   A   A   L   V   E   F   E   R   S   C   R   W   T   -
```

Supplementary Figure S4. Complete Rv3684/Cds1 amino acid sequence. (A) A list of N-terminal peptide fragments of Cds1 detected by LC-MS/MS corresponding to the correct amino acid sequence of Cds1. (**B**) The correct *rv3684/cde1* ORF includes an additional 66 nucleotides encoding 22 additional N-terminal amino acids (highlighted in yellow) in contrast to the annotation in Mycobrowser.epfl.ch. The start codon of *cds1* overlaps the stop codon of *rv3683* and both ORFs are in different coding frames. The predicted start codon (ttg) of *cds1* in Mycobrowser.epfl.ch is indicated in the box. Color code of amino acid residues: Hydrophobic - AFILMVW (red); Polar - CGHNPQSTY (green); Basic charged - K and R (pink); Acidic charged - D and E (blue).

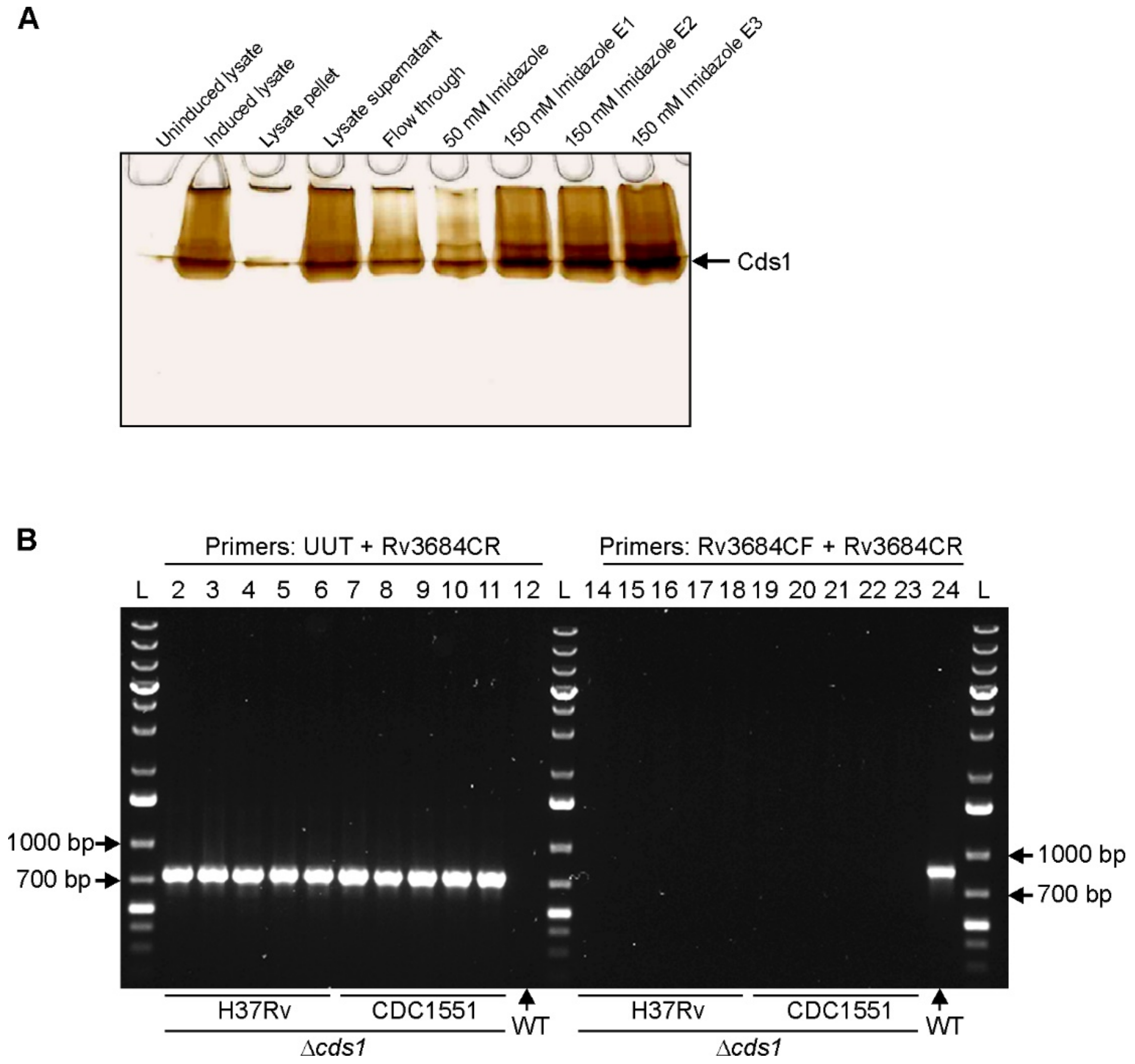

Supplementary Figure S5. In-gel BC assay of purified Cds1 and confirmation of *cds1* deletion in *Mtb* strains. (A) Eluted fractions of recombinant Cds1 were resolved on a native polyacrylamide gel and assayed for H_2S production using the in-gel BC assay. **(B)** PCR confirmation of *Mtb* H37Rv (*n* =5) and CDC1551 *cds1* knockout mutants (*n* = 5). Bands in lanes 2 to 6 (*Mtb* H37Rv) and lanes 7 to 11 (*Mtb* CDC1551) correspond to PCR amplicons (727 bp) generated using primers UUT and Rv3684CR. As the annealing site for primer UUT is only present in the disrupted *cds1*, no amplicon was generated for WT *Mtb* H37Rv (lane 12). The band in lane 24 corresponds to the PCR amplicon (819 bp) generated in WT *Mtb* H37Rv using primers Rv3684CF and Rv3684CR. The annealing site for primer Rv3684CF is present in *cds1,* but absent in the *cds1* deletion mutant and therefore no PCR amplicons were generated (lanes 14 to 23).

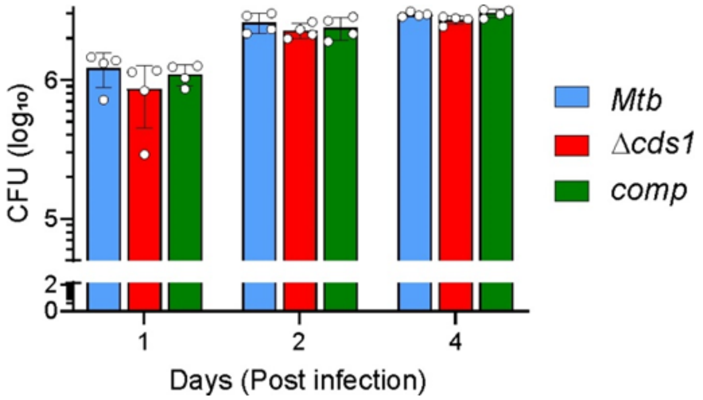

Supplementary Figure S6. Survival of *cds1*-deficient *Mtb* in macrophages. Peritoneal macrophages were obtained from C57BL/6 mice and infected with *Mtb* at an MOI ~0.2. Infected macrophages were lysed at indicated times after infection and lysates plated on 7H11 agar plates to determine bacillary burden. No statistically significant differences in cellular burden between WT *Mtb* and *Mtb Δcds1* cells were found. Data shown represents the mean ± SD for 4 replicates. Statistical analysis was performed using GraphPad Prism 8.4.3. Two-way ANOVA with Dunnett's multiple comparisons test was used to determine statistical significance.

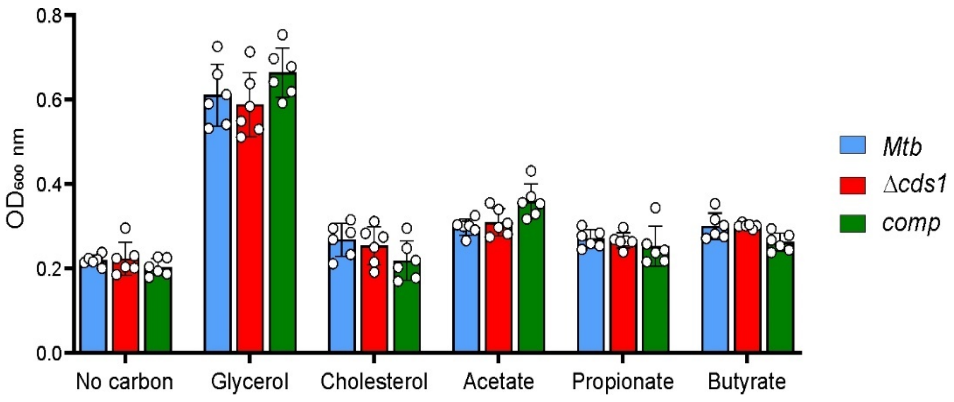

Supplementary Figure S7. *Mtb* Δ*cds1* growth in the presence of fatty acids or precursors as a single carbon source. Growth of *Mtb* strains was monitored at $OD_{600\ nm}$ using fatty acids or precursors (acetate) as a single carbon source. There were no significant growth differences between WT *Mtb* and Δ*cds1* cells. Data shown represents the mean ± SD for 6 replicates. Statistical analysis was performed using GraphPad Prism 8.4.3. Two-way ANOVA with Dunnett's multiple comparisons test was used to determine statistical significance.

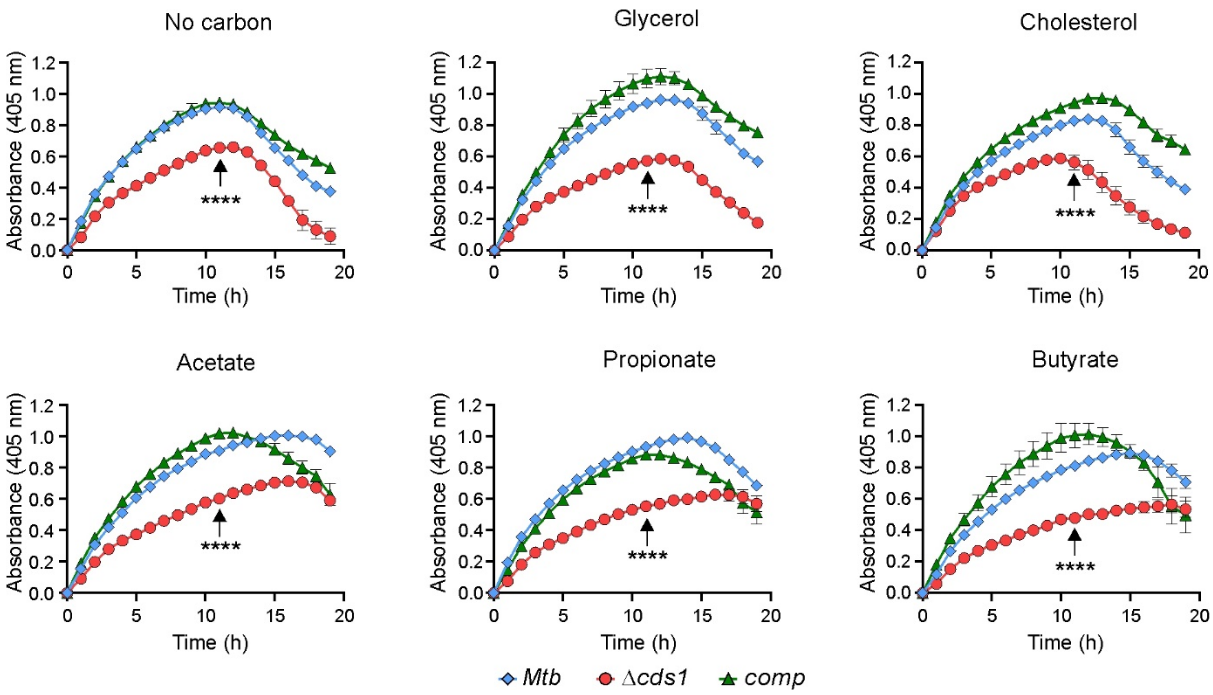

Supplementary Figure S8. ***Mtb*** **H_2S production when cultured in the presence of fatty acids or precursors as a single carbon source.** The BC assay was used to measure H_2S production of *Mtb* cultured in the presence of fatty acids or precursors (acetate) as a single carbon source. H_2S production was significantly less in Δ*cds1* cells compared to WT or complemented cells in all growth media at 12 h (vertical arrow). Data represent the mean ± SEM for 4 replicates. Statistical analysis was performed using GraphPad Prism 8.4.3. Two-way ANOVA with Dunnett's multiple comparisons test was used to determine statistical significance. ****$P < 0.0001$.

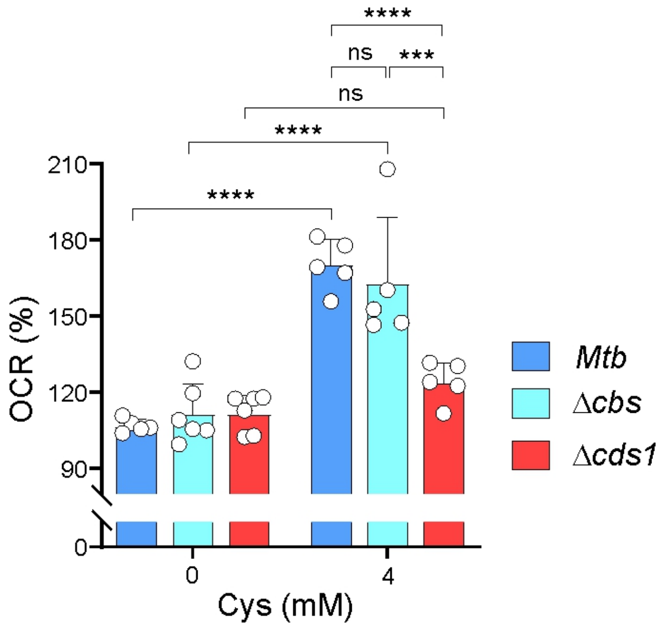

Supplementary Figure S9. Role of Cbs (Rv1077) in *Mtb* respiration. To determine whether Cbs (Rv1077) is important for respiration in *Mtb*, the oxygen consumption rates (OCR) of WT *Mtb*, Δ*cbs* and Δ*cds1* were measured using an Agilent Seahorse XFe96 Analyzer. %OCR was measured in *Mtb* strains grown in medium containing 0 or 4 mM Cys. Cbs does not appear to play a significant role in *Mtb* respiration under these conditions. Note the role of Cds1 in respiration under the same conditions. One representative experiment is shown. The experiment was repeated twice. Data represent the mean ± SD for 5 – 6 replicates. Statistical analysis was performed using GraphPad Prism 8.4.3. Two-way ANOVA with Tukey's multiple comparisons test was used to determine statistical significance. ***P < 0.001, ****P < 0.0001, ns – statistically non-significant.

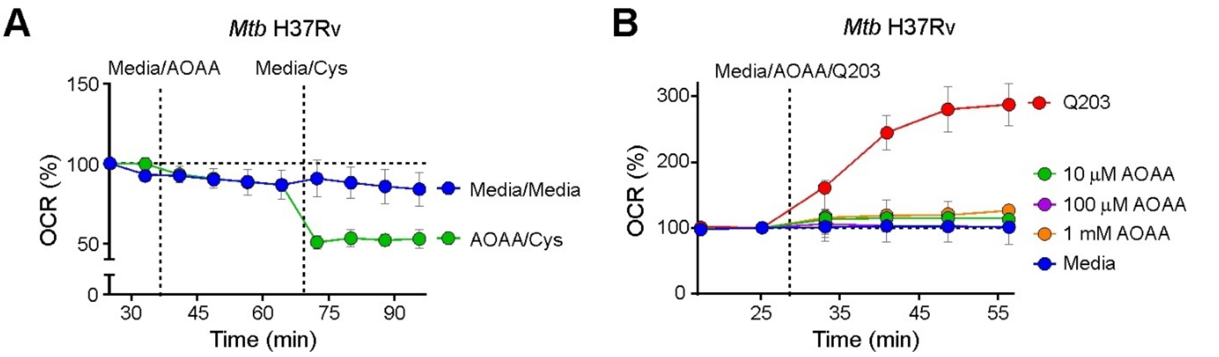

Supplementary Figure S10. AOAA inhibits Cys-mediated increases in *Mtb* respiration. **(A)** %OCR of *Mtb* following sequential injection of 1 mM AOAA and 1 mM Cys, or media as a control. Whereas Cys increases respiration (Figure 5b), which can be inhibited by AOAA (Figure 5d), the data here suggest that pre-treatment of cells with AOAA affects metabolism by targeting PLP-dependent enzymes, but not respiration. Here, following Cys addition, AOAA-treated cells are unable to respond homeostatically to Cys-generated H_2S that normally stimulates respiration. Under these conditions, AOAA inhibits numerous PLP-dependent enzymes as well as Cds1, which collectively are unable to maintain respiratory homeostasis. **(B)** %OCR of *Mtb* grown in Cys-free medium and exposed to different concentrations of AOAA, showing that AOAA alone does not alter OCR. The 100 µM AOAA data (magenta line) is obscured by other data points. Data are representative of 2-3 independent experiments, showing mean ± SD for n = 6 – 8 replicates. Statistical analysis was performed using GraphPad Prism 8.4.3. The anti-TB drug Q203 (300 x MIC_{50}) that stimulates *Mtb* respiration was used as positive control in **(B)**.

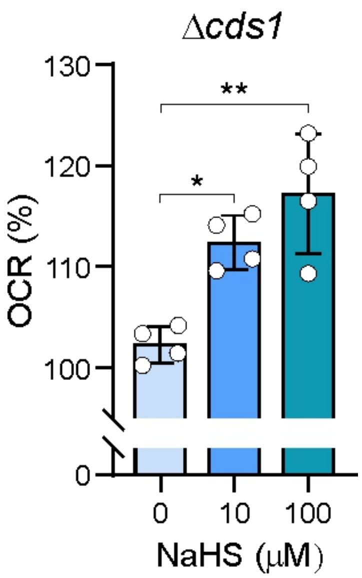

Supplementary Figure S11. Exogenous H_2S reverses the respiratory defect in *Mtb* Δ*cds1* cells. Exposing *Mtb* Δ*cds1* cells to different concentrations (0, 10, 100 μM) of exogenous H_2S (NaHS) increases bacillary %OCR. Data is representative of two independent experiments, showing mean ± SD for $n = 4$ replicates. Statistical analysis was performed using GraphPad Prism 8.4.3. Two-way ANOVA with Dunnett's multiple comparisons test was used to determine statistical significance. *$P < 0.05$, **$P < 0.01$.

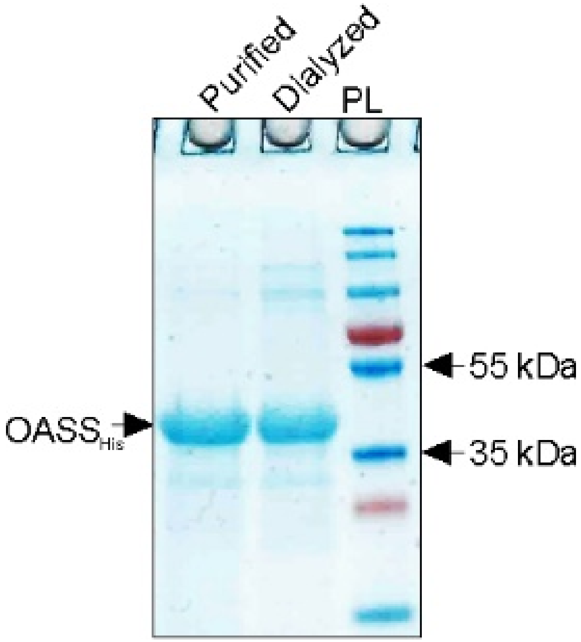

Supplementary Figure S12. SDS-PAGE of purified *O*-acetylserine sulfhydrylase (OASS). Recombinant OASS containing an N-terminal 6xHis tag was expressed from plasmid pET28b-EhOASS and purified from *E. coli* lysates as described previously[3].

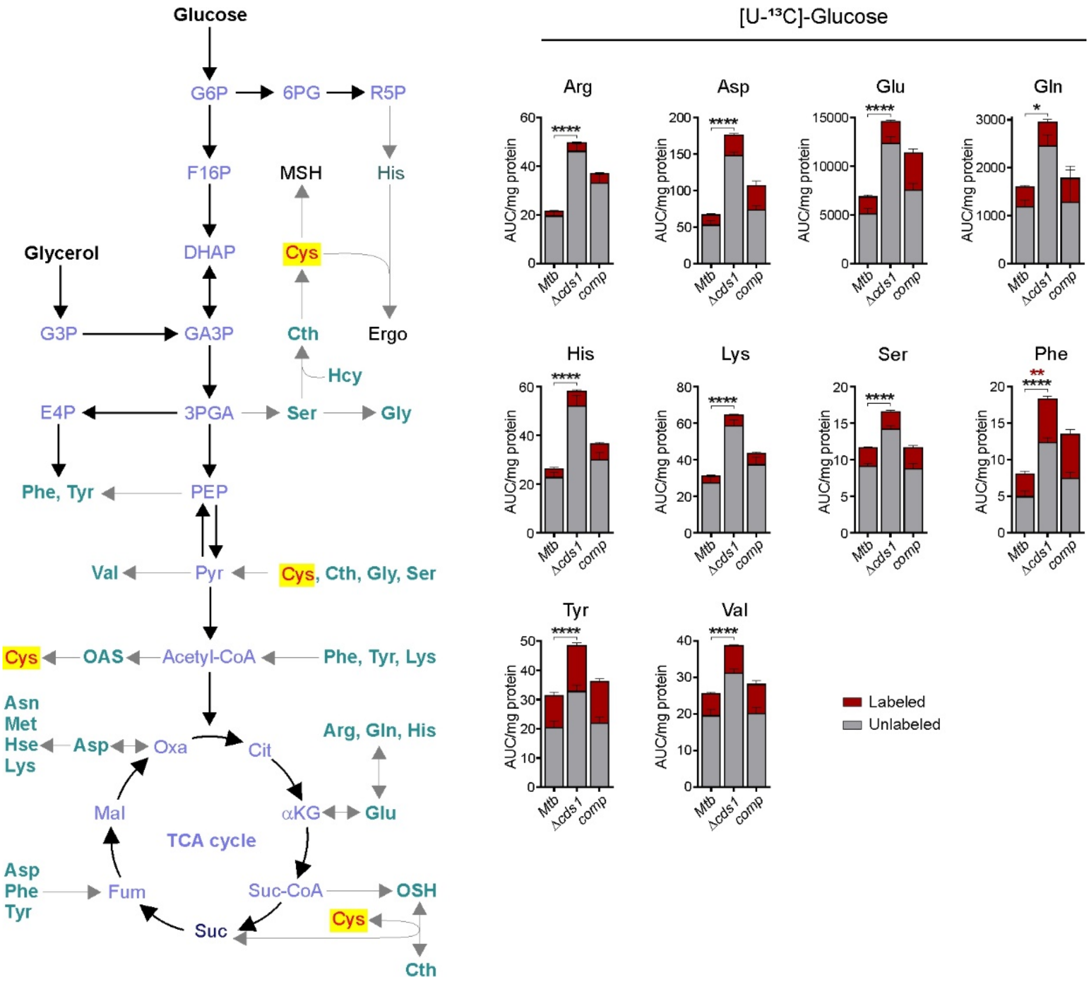

Supplementary Figure S13. Cds1 regulates amino acid metabolism in *Mtb*. *Mtb* strains were cultured in 7H9 medium with [U-^{13}C]-Glucose (0.2%) followed by LC-MS/MS analysis. Total abundance of ^{13}C-labeled (Red) and unlabeled (Gray) amino acids are indicated. Representative experiments are shown; the experiment was repeated twice. Data shown represents the mean ± SEM for 3 – 5 biological replicates. Statistical analysis was performed using GraphPad Prism 8.4.3. Two-way ANOVA with Dunnett's multiple comparisons test was used to determine statistical significance. *P < 0.05, **P < 0.01, ****P < 0.0001.

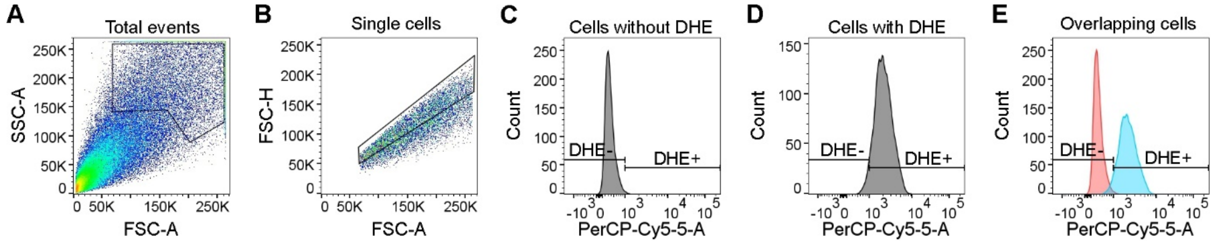

Supplementary Figure S14. Gating strategy for detection of DHE-positive *Mtb* cells for measuring ROI. ROI level in *Mtb* strains was measured using the dihydroethidium ROI-sensing dye (DHE, PerCP-Cy5.5). Gating strategy for **(A)** total events (100,000 cells), **(B)** single cells, **(C)** unstained cells without DHE, **(D)** DHE-stained cells, **(E)** overlapping histogram of (C) and (D) to show the population of DHE-positive and -negative cells.

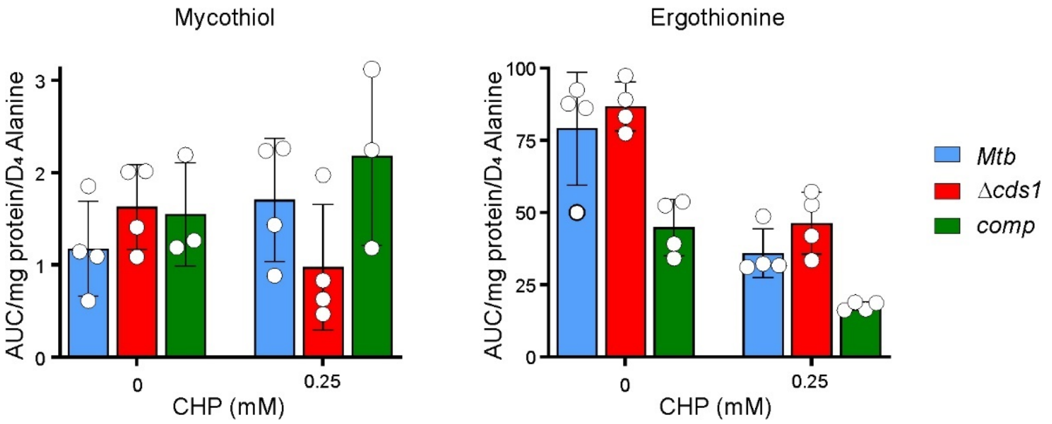

Supplementary Figure S15. Mycothiol and ergothioneine levels in *Mtb* after exposure to CHP. Mycothiol and ergothioneine were quantified in *Mtb* strains using LC-MS/MS after exposure to CHP for 16 h. No significant differences were observed between WT *Mtb* and *Mtb Δcds1* cells. Data shown represents the mean ± SEM for *n* = 3-4 replicates. Statistical analysis was performed using GraphPad Prism 8.4.3. Two-way ANOVA with Dunnett's multiple comparisons test was used to determine statistical significance.

Supplementary Table S1. Bacterial strains used in this study.

Strain	Description	Source
M. tuberculosis H37Rv	Wild type (wt)	ATCC
M. bovis BCG Pasteur	Vaccine strain	ATCC
M. bovis	Wild type	ATCC
M. tuberculosis TKK-01-0027	Clinical drug susceptible *Mtb* strain (DS27)	Alex Pym, AHRI
M. tuberculosis TKK-01-0047	Clinical drug susceptible *Mtb* strain (DS47)	Alex Pym, AHRI
M. tuberculosis TKK-01-0035	Clinical multi-drug resistant *Mtb* strain (MDR35)	Alex Pym, AHRI
M. tuberculosis TKK-01-0001	Clinical multi-drug resistant *Mtb* strain (MDR01)	Alex Pym, AHRI
M. tuberculosis Δ*cbs*	*cbs* deletion mutant (Δ*cbs*) in H37Rv; Hyg^R	This study
M. tuberculosis Δ*cds1*	*cds1* deletion mutant (Δ*cds1*) in H37Rv; Hyg^R	This study
M. tuberculosis Δ*cds1::hsp*$_{60}$*-cds1*	*cds1* complement (*comp*) of Δ*cds1*; Hyg^R and Kan^R	This study
M. tuberculosis CDC1551	Wild type (wt)	ATCC
M. tuberculosis Tn::rv3682	*rv3682* transposon insertion mutant in CDC1551; Kan^R	John Hopkins University, School of Medicine, TARGET
M. tuberculosis Tn::rv3683	*rv3683* transposon insertion mutant in CDC1551; Kan^R	John Hopkins University, School of Medicine, TARGET
M. tuberculosis Δ*cydAB*	*cydAB* deletion mutant (Δ*cydAB*) in *Mtb* H37Rv; Hyg^R	Helena Boshoff[12], NIAID
M. smegmatis mc^{2}155	Wild type (wt)	ATCC
M. smegmatis wt$_p$*-cds1*	*rv3682-rv3683-cds1* under control of the *Mtb* native promoter (*wt*$_p$) in mc^{2}155	This study
M. smegmatis hsp$_{60}$*-cds1*	*rv3682-rv3683-cds1* under control of the *hsp*$_{60}$ promoter in mc^{2}155	This study

Supplementary Table S2. Plasmids used in this study.

Vector/construct	Relevant genotype and properties	Source
pMV261	*E. coli* Mycobacterium *shuttle vector, hsp* hsp_{60}, *ColE1/pAL500 oriM,* Kan^R	William R. Jacobs Jr. (Albert Einstein College of Medicine)
pET28b-OASS	Construct encoding N-terminally 6xHis-tagged EhOASS (O-acetylserine sulfhydrylase from *Entamoeba histolytica*)	Alessandro Giuffrè (CNR Institute of Molecular Biology and Pathology, Rome, Italy)
cds1 phasmid	*cds1::res–hyg–res*	Michelle Larsen (Albert Einstein College of Medicine)
cbs phasmid	*cbs::res–hyg–res*	Michelle Larsen (Albert Einstein College of Medicine)
pMV261::hsp_{60}-*cds1*	The *cds1* open reading frame cloned under the control of the *hsp60* promoter cloned into pMV261	This study
pMV261::wt_p-*cds1*	The *rv3682-rv3683-cds1* open reading frames containing the native promoter (wt_p) cloned into pMV261	This study
pET15b	amp^r, *E. coli* vector used for production of his-tag fused proteins	Novagen
pET15b-*cds1*	*Mtb cds1* ORF cloned into pET15b	This study

Supplementary Table S3. Oligonucleotides used in this study.

Oligonucleotide	Sequence (5' → 3')	Description
Rv3684F	TATGGATCCTATGAGCGGCGGGGCCTGTATC	*cds1* forward primer for pMV261 subcloning, *Bam* HI
Rv3684R	GTTATCGATTAGGCTGCGGACCGCGATAC	*cds1* reverse primer for pMV261 subcloning, *Cla* I
ponABCF	TAAGGATCCAAGGTAGTCCGACCACGAAAC	*rv3682*, *rv3683* and *cds1* forward primer, *Bam* HI
ponABCR	ATAATCGATCTACCAAGCTGCGCCACAC	*rv3682*, *rv3683* and *cds1* reverse primer, *Cla* I
Rv3684CF	GAACCCAATGAACTATCTGAC	Forward primer for Δ*cds1* confirmation
Rv3684CR	GCATAGCGCATAGAGGAA	Reverse primer for Δ*cds1* confirmation
UUT	GATGTCTCACTGAGGTCTCT	"Universal uptag" primer for Δ*cds1* confirmation
Rv3684CEF	AATAATCATATGTTGAGCGGCGGGGCCT	*cds1* forward primer for pET15b subcloning, *Nde* I
Rv3684CER	AATAATGGATCCTCACGTCCATCGACAG	*cds1* reverse primer for pET15b subcloning, *Bam* HI
Rv1077CF	GGTCGACTATCGGTTGATT	Forward primer for Δ*rv1077* confirmation
Rv1077CR	ACATTGCGTTTATCCTCACT	Reverse primer for Δ*rv1077* confirmation

Supplementary Table S4. *Mtb* H37Rv enzymes putatively involved in sulfur-containing amino acid biosynthesis, H_2S production or sulfur metabolism.

	**Enzymes capable of Sulfide reactions**	***Mtb* Locus**	**Gene Product**	**Annotated Pathway/Function**	**Catalytic Activity**	***Ref.***
1	Probable cystathionine γ-synthase/O-succinyl homoserine sulfhydrylase	Rv0391	MetZ	Methionine Biosynthesis/ Probable Cystathionine γ-synthase	*O*-succinylhomoserine → homocysteine	4
2	Cysteine synthase	Rv0848	CysK2	Cysteine Biosynthesis	(1) *O*-phospho-L-serine → *S*-sulfocysteine (2) *O*-phospho-L-serine + H_2S → L-cysteine + phosphate	5
3	Cystathionine β-synthase	Rv1077	CBS	Cysteine Biosynthesis/Serine sulfhydrase/ Transulfuration Pathway	(1) homocysteine + serine → cystathionine (2) cysteine + homocysteine → cystathionine + H_2S	6
4	Cystathionine γ-synthase/cystathionine γ-lyase	Rv1079	MetB	Methionine Biosynthesis/Probable Cystathionine γ-synthase/ Transulfuration Pathway	(1) *O*-succinyl-L-homoserine + L-cysteine -> cystathionine + succinate (2) cystathionine → α-ketobutyrate +NH_3	4
5	Cysteine synthase	Rv1336	CysM	Cysteine Biosynthesis	*O*-phospho-L-serine + CysO-SH → CysO-Cys + PO_4^{3-}	5
6	Cysteine synthase	Rv2334	CysK1	Cysteine Biosynthesis	*O*-acetyl-L-serine + H_2S → L-cysteine + acetate	5
7	Ferredoxin-dependent sulfite reductase	Rv2391	SirA	Sulfate Assimilation	SO_3^{2-} → S^{2-}	7
**8**	Methionine synthase	Rv3340	MetC	Methionine Biosynthesis/Probable O-acetyl homoserine sulfhydrylase	(1) O-acetyl-L-homoserine + methanethiol → L-methionine + acetate (2) *O*-acetyl-L-homoserine + H_2S ↔ L-homocysteine + acetate	8
**10**	Probable cysteine desulfhydrase/ cysteine synthase /cystathionine γ-lyase?	Rv3684	Cysteine synthase or lyase	Unclassified/Cys Metabolism/ Transulfuration Pathway?	Cysteine → pyruvate + H_2S + NH_3	9
**11**	Probable cysteine desulfurase	Rv3025 c	iscS	Carbon Sulfur Lyase	[sulfur carrier]-H + L-cysteine- [sulfur carrier]-SH + L-alanine	10, 11

References

1. Robert X, Gouet P. Deciphering key features in protein structures with the new ENDscript server. *Nucleic Acids Research* **42**, W320-324 (2014).

2. Notredame C, Higgins DG, Heringa J. T-Coffee: A novel method for fast and accurate multiple sequence alignment. *Journal of Molecular Biology* **302**, 205-217 (2000).

3. Forte E*, et al.* The Terminal Oxidase Cytochrome bd Promotes Sulfide-resistant Bacterial Respiration and Growth. *Sci Rep* **6**, 23788 (2016).

4. Parish T, Gordhan BG, McAdam RA, Duncan K, Mizrahi V, Stoker NG. Production of mutants in amino acid biosynthesis genes of *Mycobacterium tuberculosis* by homologous recombination. *Microbiology* **145 (Pt 12)**, 3497-3503 (1999).

5. Schnell R, Sriram D, Schneider G. Pyridoxal-phosphate dependent mycobacterial cysteine synthases: Structure, mechanism and potential as drug targets. *Biochimica et Biophysica Acta* **1854**, 1175-1183 (2015).

6. Singhal A*, et al.* Regulation of homocysteine metabolism by *Mycobacterium tuberculosis* S-adenosylhomocysteine hydrolase. *Sci Rep* **3**, 2264 (2013).

7. Hatzios SK, Bertozzi CR. The regulation of sulfur metabolism in Mycobacterium tuberculosis. *PLoS Pathogens* **7**, e1002036 (2011).

8. Yin J*, et al.* Expression, purification and preliminary crystallographic analysis of O-acetylhomoserine sulfhydrylase from *Mycobacterium tuberculosis*. *Acta Crystallographica Section F, Structural biology and crystallization communications* **67**, 959-963 (2011).

9. Burns-Huang K, Mundhra S. *Mycobacterium tuberculosis* cysteine biosynthesis genes mec+-cysO-cysM confer resistance to clofazimine. *Tuberculosis (Edinburgh, Scotland)* **115**, 63-66 (2019).

10. Singh A*, et al. Mycobacterium tuberculosis* WhiB3 responds to O_2 and nitric oxide via its [4Fe-4S] cluster and is essential for nutrient starvation survival. *Proc Natl Acad Sci U S A* **104**, 11562-11567 (2007).

11. Schwartz CJ, Djaman O, Imlay JA, Kiley PJ. The cysteine desulfurase, IscS, has a major role in in vivo Fe-S cluster formation in *Escherichia coli*. *Proc Natl Acad Sci U S A* **97**, 9009-9014 (2000).

12. Arora K*, et al.* Respiratory flexibility in response to inhibition of cytochrome C oxidase in *Mycobacterium tuberculosis*. *Antimicrob Agents Chemother* **58**, 6962-6965 (2014).
